
JOURNAL INFORMATION
==============================
NLM Title Abbreviation: J Child Orthop
Journal ID: J Child Orthop
NLM Journal ID: 101313582

Journal of Children's Orthopaedics

ISSN: 1863-2521
EISSN: 1863-2548
Publisher: SAGE Publications

ARTICLE INFORMATION
==============================
PMCID: PMC4661147
DOI: 10.1007/s11832-015-0708-2
Article ID: 708
Article version: 1
Subjects: Acknowledgement

Acknowledgement to reviewers 2015

Electronic publication date: 2015 Nov 19
Print publication date: 2015 Dec
Volume: 9
Issue: 6
First page: 509
Last page: 510
Copyright: © The Author(s) 2015
License: This article is distributed under the terms of the Creative Commons Attribution 4.0 International License (http://creativecommons.org/licenses/by/4.0/), which permits unrestricted use, distribution, and reproduction in any medium, provided you give appropriate credit to the original author(s) and the source, provide a link to the Creative Commons license, and indicate if changes were made.
License URL: https://creativecommons.org/licenses/by/4.0/

issue-copyright-statement: © The Author(s) 2015

==============================

The Editors-In-Chief would like to thank the following reviewers for their continuous support during the year of 2015:

Franck Accadbled
Michael D. Aiona
Zaid Al-Aubaidi
Javier Albinana
Cristina Alves
Antonio Andreacchio
David D. Aronsson
Elhanan Bar-On
Jeremy Bauer
James Beaty
Michael J. Bell
Mohan V. Belthur
Michael K. D. Benson
Ernesto Bersusky
Victor Bialik
Rainer G. Biedermann
John G. Birch
Robert D. Blasier
Stephanie Boehm
Thomas Boeni
Silvio Boero
Christopher F. Bradish
Daniel Brandenberger
Silviu Brill
Reinald Brunner
Carlo Camathias
Federico Canavese
Norris C. Carroll
Pablo Castañeda
Anthony Catterall
Tae-Joon Cho
Franck Chotel
Leonel Copeliovitch
Aurélien Courvoisier
Juan Carlos Couto
Alvin H. Crawford
Robert J. Cummings
Peter J. Cundy
Jaroslaw Czubak
Charles d'Amato
Aina J. Danielsson
Luciano Dias
John P. Dormans
Denis S. Drummond
Deborah Eastwood
Eric W. Edmonds
John Emans
Thomas Erb
G. Ulrich Exner
Eli Ezra
Guy Fabry
Francois Fassier
David S. Feldman
Frank Fitoussi
Elvira Flikweert
Edilson Forlin
Niklaus F. Friederich
Bruno Fuchs
Patricia M. B. Fucs
Toshio Fujii
Julia F. Funk
Rudolf Ganger
Yael Gelfer
Ismat Ghanem
Werner Girsch
Uri Givon
Reinhard Graf
H. Kerr Graham
Alfred D. Grant
Franz Grill
Richard H. Gross
Gunnar Hägglund
Chiaki Hamanishi
Carol Hasler
Martin Haug
Dorian Hauke
Shlomo Hayek
Hanne B. Hedin
Bernhard Heimkes
Anna K. Hell
Yoram Hemo
John A. Herring
Benton E. Heyworth
Sevan Hopyan
Gamal Hosny
James B. Hunter
Ivan Hvid
Ernesto Ippolito
Roger Jawish
Charles E. Johnston
Stanley Jones
Benjamin Joseph
Jean-Luc Jouve

Andre Kaelin
Lori Karol
Hidehiko Kawabata
Derek M. Kelly
David Keret
Joseph I. Krajbich
Ruediger Krauspe
Andreas H. Krieg
Ken N. Kuo
Erica Lamprecht
Lennart A. Landin
Pierre Lascombes
David Lebel
Wallace Lehman
David G. Little
Randall Loder
John E. Lonstein
Scott J. Luhmann
Nicolas Lutz
William G. Mackenzie
Eran Maman
Johannes Mayr
Terence McGuire
Charles T. Mehlman
A. Ludwig Meiss
Carlo Milani
Freeman Miller
Bjarne Moeller-Madsen
Guy Molenaers
Jose Morcuende
Vincent Mosca
Scott J. Mubarak
Kishore Mulpuri
Marcus Mumme
Yasuharu Nakashima
Marek Napiontek
Cornelia Neuhaus
Yrjänä Nietosvaara
Andrej Nowakowski
Iulian Nusem
Avi Ohry
Hakan Omeroglu
Norman Y. Otsuka
Dror Ovadia
Klaus Parsch
Peter Pizzutillo
Jan Poul
Thomas Pressel
Tamir Pritsch
George T. Rab
Tariq Rahman
Leonhard E. Ramseier
Andreas Roposch
Yishai Rosenblatt
Erich Rutz
Peter B. Salamon
Wudbhav N. Sankar
Dietrich Schlenzka
Perry L. Schoenecker
Eitan N. Segev
Dalia A. Sepúlveda
Sheldon R. Simon
Theddy Slongo
Hae Ryong Song
Bernhard M. Speth
Paul D. Sponseller
Carl L. Stanitski
Walter M. Strobl
Daniel Studer
Michael D. Sussman
Marek Synder
Terje Terjesen
Tim Theologis
Stephen J. Tredwell
Christian Tschauner
Cosimo Tudisco
Anja Van Campenhout
Johannes A. van der Sluijs
Andrew Wainwright
Stewart Walsh
John H. Wedge
Dennis S. Weiner
Stuart L. Weinstein
Dennis R. Wenger
Bettina Westhoff
Roger F. Widmann
R. Baxter Willis
Thomas Wirth
James G. Wright
Moshe Yaniv
Reinhard D. Zeller

Open Access

This article is distributed under the terms of the Creative Commons Attribution 4.0 International License (http://creativecommons.org/licenses/by/4.0/), which permits unrestricted use, distribution, and reproduction in any medium, provided you give appropriate credit to the original author(s) and the source, provide a link to the Creative Commons license, and indicate if changes were made.

